# CAEFRER-YOLO11: An Adaptive Feature Refinement-Based Hybrid Model and Dataset for Weld Defect Detection

**DOI:** 10.3390/ma19214534

**Published:** 2026-10-28

**Authors:** Adem Dilbaz, Ilker Ali Ozkan

**Affiliations:** 1 Sahin Tanker Limited Company, Asagipinarbasi Organized Industrial Zone, Konya 42160, Turkey; 2 Department of Computer Engineering, Faculty of Technology, Selcuk University, Konya 42130, Turkey; ilkerozkan@selcuk.edu.tr

**Keywords:** CAEFRER-YOLO11, deep learning, Eigen-CAM, RT-DETR, welding defect detection, YOLO11

## Abstract

**Highlights:**

- CAEFRER-YOLO11 combines YOLO11 feature extraction, the CAEFRER framework, an RT-DETR-based decoder, and a detection head.
- The proposed framework enhances the detection of complex and small defects.
- A hybrid HWD dataset is developed to improve class balance using real-world images.
- Systematic evaluations analyze YOLO11, RT-DETR, and the proposed CAEFRER-YOLO11.
- Eigen-CAM and region masking assess the spatial fidelity of defect localizations.

**Abstract:**

Weld quality inspection is essential for ensuring structural integrity, product reliability, and manufacturing safety in industrial applications. However, conventional visual inspection methods can be time-consuming, operator-dependent, and prone to overlooking subtle weld defects. YOLO11-based detectors employ feature extraction and aggregation mechanisms that may be insufficient to reliably distinguish informative from ambiguous feature responses under challenging industrial imaging conditions, making the localization of small and subtle weld defects difficult. This study proposes the CAEFRER (Context-Aware Enhanced Feature Refinement Encoder with Re-parameterization) framework for weld defect detection and employs post hoc explainability analysis to investigate whether model predictions correspond to defect-relevant regions. The proposed CAEFRER-YOLO11 introduces an adaptive feature map refinement strategy that integrates feature regeneration and re-parameterization within a hybrid architecture. The proposed Hybrid Weld Defect (HWD) dataset comprises approximately 3500 images of ST37 steel, Hardox 450 steel, and aluminum welds produced using metal active gas (MAG), metal inert gas (MIG) and submerged arc welding (SAW) processes. Experiments were conducted on the HWD and the Weld Defect Detection (WDD) datasets using YOLO- and DETR-based architectures. On the HWD dataset, the proposed model incorporating the CAEFRER framework achieved improved performance, with ${\mathrm{mAP}}_{50–95}$ gains of up to 2.09 percentage points (pp) over the baseline YOLO11-L and 0.75 pp over RT-DETR-L. Furthermore, Eigen-CAM-based activation map analysis demonstrated that CAEFRER-YOLO11 trained on the proposed dataset focused more accurately on the actual weld defect regions. Overall, the proposed CAEFRER framework and HWD dataset contribute to improved weld defect detection performance and enable more reliable explainability by generating activation maps that are better aligned with the true defect regions.

## 1. Introduction

### 1.1. Background

Welding is a key joining process in industrial manufacturing, where defects can lead to mechanical degradation, structural failure, and significant economic losses [1,2]. Therefore, reliable inspection systems are essential components of industrial quality assurance frameworks [3]. Traditional inspection methods rely on manual visual assessment and non-destructive testing techniques such as radiographic imaging, ultrasonic evaluation, and magnetic particle inspection [4]. Although widely used, these approaches are time-consuming, require expert knowledge, and are prone to operator-dependent variability [5]. In addition, manual inspection is limited by fatigue and inconsistent evaluations in high-throughput industrial environments [6]. These limitations underscore the urgent industrial need for efficient, reliable, and automated weld defect detection systems to improve product quality and production efficiency [7]. To address these challenges, YOLO-based object detectors have been widely applied to weld defect inspection due to their favorable balance between detection accuracy and computational efficiency [8,9]. Recent YOLO variants, including YOLO11, have indicated improved detection capability for small and complex defect regions [10,11]. However, despite these advancements, most YOLO-based detection frameworks rely on static feature fusion mechanisms that propagate unreliable feature responses without explicitly considering feature reliability under challenging industrial imaging conditions such as spatter, blur, low contrast, and illumination variations.

Transformer-based detectors use global self-attention as an alternative to CNN-based approaches. DETR reformulates object detection as a set prediction problem but has limitations in convergence and small-object detection. RT-DETR further improves the architecture for efficient detection, although computational efficiency remains important for industrial deployment [12]. At the feature level, multi-scale feature maps provide multi-scale visual information for object detection but may suffer from degradation under noisy industrial imaging conditions, particularly for small and subtle defects [13].

### 1.2. Motivation

Industrial welding defects such as incomplete fusion, lack of penetration, porosity, and undercut may cause mechanical weakness and production downtime if not accurately detected [14,15]. Nevertheless, subtle textures, low contrast, ambiguous boundaries, and imaging artifacts remain challenging for reliable weld defect detection [16]. Many existing studies report high overall detection accuracy but provide limited analysis of robustness across difficult defect categories. Furthermore, existing feature aggregation strategies lack adaptive mechanisms for suppressing unreliable activations under noisy industrial conditions, potentially affecting localization and classification performance. In particular, inadequate weld leg length, which is associated with weld geometry and dimensional conformity, can directly affect the load-bearing capacity of welded joints, underscoring the importance of reliable detection of weld-related anomalies [17].

Another important consideration is the limited investigation of model interpretability. Existing welding inspection studies often emphasize quantitative detection metrics, while comparatively less attention is given to examining whether model attention consistently corresponds to physically meaningful defect regions during inference [18]. In this context, Eigen-CAM (Eigen Class Activation Map) provides a gradient-free approach for visualizing spatial activation patterns by leveraging principal component analysis (PCA) to identify dominant patterns in activation feature maps and examine the regions emphasized by the model during prediction [19].

In addition, transformer-based detectors such as DETR have shown promising capability in modeling complex defect patterns, but their robustness under class-imbalanced and challenging industrial conditions may differ substantially from YOLO-based detectors [20,21].

Motivated by these limitations, this study proposes CAEFRER-YOLO11, a model incorporating a reliability-aware feature refinement framework that iteratively estimates feature reliability and selectively enhances informative defect representations through adaptive refinement mechanisms.

**Figure 1:**
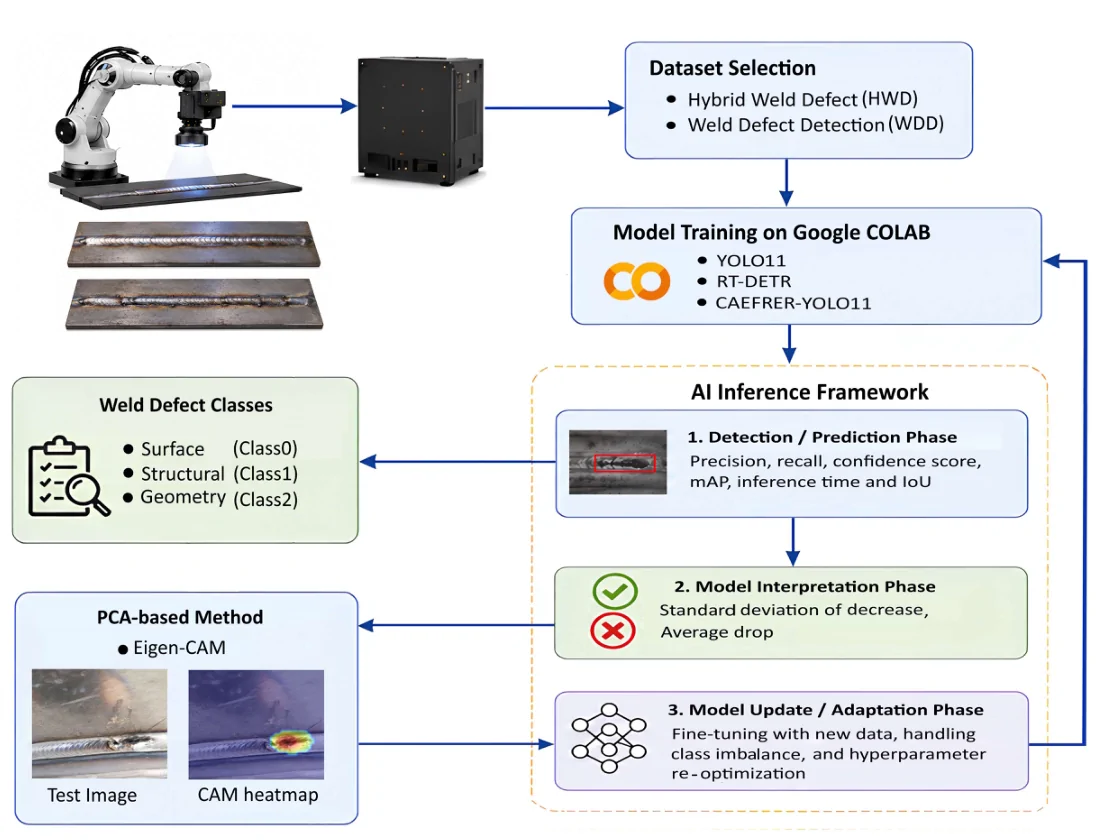
Workflow of the weld defect detection pipeline, including all evaluated YOLO- and RT-DETR-based models.

Figure 1 illustrates the comparative weld defect detection system, including YOLO11, RT-DETR, and the proposed model, evaluated on the HWD and WDD datasets with Eigen-CAM analysis. By integrating the proposed CAEFRER framework into the detection pipeline, the system aims to improve feature reliability, enhance detection robustness, and achieve more accurate localization of weld defects, particularly small defects smaller than $32^{2}$ pixels, in industrial welding environments.

### 1.3. Contributions

Based on the limitations of existing weld defect detectors under challenging industrial imaging conditions, this study introduces a comprehensive model for weld defect analysis. The main contributions of this work are summarized as follows:

- A Re-parameterized Feature Refinement-Based Hybrid Architecture (CAEFRER): This study proposes an integrated reliability-aware feature refinement approach within a hybrid detection architecture that combines the YOLO11 backbone and neck with an RT-DETR-based decoder to suppress unreliable feature responses under challenging industrial imaging conditions. The proposed CAEFRER framework incorporates adaptive feature regeneration to reinforce defect-relevant representations and improve spatial precision in defect localization. In addition, RepVGG-B0-based structural re-parameterization is incorporated to enhance multi-scale feature modeling while preserving inference efficiency. The RT-DETR-based decoder replaces the original YOLO11 detection head.
- Hybrid Weld Defect Dataset: The HWD dataset was developed by integrating publicly available Kaggle images with self-collected industrial aluminum weld images to improve data diversity, increase sample size, and better represent real industrial inspection conditions. The resulting benchmark supports more reliable evaluation of detection robustness across multiple weld defect categories.
- Comprehensive evaluation of state-of-the-art detectors: YOLO11, RT-DETR, and the proposed CAEFRER-YOLO11 are systematically evaluated under identical experimental conditions to investigate the behavior of YOLO- and transformer-based architectures in challenging industrial defect detection scenarios. Beyond standard performance metrics, convergence, training stability, and class-wise performance are analyzed to assess localization robustness and generalization under different dataset conditions.
- Explainability-driven defect analysis: Eigen-CAM is integrated for the comparative evaluation of weld defect detection in CAEFRER-YOLO11, YOLO11, and RT-DETR by incorporating high-activation region masking ($M_{1}$), background-region masking ($M_{2}$), weighted heatmap aggregation, and PCA.

## 2. Related Work

Welding defects in this study are categorized into three main groups: surface defects, structural defects, and geometric defects. According to Eren, among these, structural defects, such as lack of fusion and incomplete penetration, are particularly important because they can compromise weld integrity and may contribute to premature failure under service conditions [22]. Although numerous datasets have been introduced for weld defect analysis, as highlighted by Block et al. and Zhang et al. [23,24], the diversity and complexity of industrial welding environments still require datasets with improved representativeness and annotation quality. Numerous AI-based approaches, particularly the YOLO-based variants proposed by Wang et al. and Luo et al. [25,26], have achieved mAP improvements of approximately 2–8% over baseline detection methods. By improving multi-scale feature representation and small-object detection capability, the methods developed by Rappe et al. and Celles et al. [27,28] have demonstrated enhanced performance in major welding inspection tasks, including crack detection and lack of fusion analysis. However, most existing methods still rely on static feature aggregation mechanisms and remain vulnerable to industrial imaging artifacts such as background clutter, reflections, uneven illumination, and low-contrast textures, making subtle defects difficult to localize reliably. These limitations motivate the development of more reliable feature refinement strategies for robust weld defect detection.

RT-DETR-based detectors have emerged as a competitive alternative to CNN-based architectures by using global self-attention for feature modeling. DETR further formulated object detection as a set prediction problem within an end-to-end Transformer-based framework. However, as noted by Meng et al. [29], the original DETR suffers from slow training convergence and relatively limited performance in detecting small objects, which can be challenging for applications involving small and subtle defects. Nonetheless, González Hernández et al. [30] demonstrated that RT-DETR can achieve competitive detection performance and computational efficiency relative to YOLO-based detectors, highlighting its potential for real-time object detection applications. Similarly, Dilbaz [31] conducted a comparative evaluation of YOLOv10 and RT-DETR architectures for weld defect detection under identical experimental conditions, demonstrating that RT-DETR-X achieved the highest ${\mathrm{mAP}}_{50–95}$ (45.8%), whereas YOLOv10-X obtained the highest precision (76.8%). These findings highlight the complementary strengths of YOLO- and Transformer-based detectors in automated weld defect inspection. Although transformer-based detectors show strong capability in modeling complex feature interactions, their robustness under noisy industrial conditions and for subtle defect categories remains insufficiently explored. Moreover, both YOLO- and transformer-based detectors are still susceptible to unreliable feature responses induced by industrial imaging artifacts, particularly when detecting small, ambiguous welding defects.

As emphasized by Zhang et al. [32], Explainable Artificial Intelligence (XAI) has become increasingly important for trustworthy industrial inspection systems, particularly in safety-critical manufacturing environments. López et al. stated that CAM-based visualization methods, such as Grad-CAM, generate class-discriminative localization maps that enable the interpretation of detector attention behavior [33]. Wu et al. have also employed these approaches in weld defect detection to visualize the spatial activation patterns of deep learning models and examine whether the highlighted regions correspond to defect-relevant areas in weld images [34]. Motivated by these advances, Eigen-CAM was selected in this study because it generates activation maps without relying on gradient computation, making it directly applicable to both YOLO11 and the transformer-based RT-DETR for a fair and consistent comparative analysis. Recent studies by Nikam et al. and Liu et al. have employed CAM to investigate whether deep learning models focus on defect-relevant regions in welding images, providing qualitative insight into their spatial decision patterns [35,36]. Accordingly, Eigen-CAM is employed in this study to qualitatively evaluate and compare the localization behavior and robustness of CAEFRER-YOLO11, YOLO11, and RT-DETR under different industrial imaging conditions.

## 3. Materials and Methods

### 3.1. The Architecture of YOLO

YOLO is a state-of-the-art single-stage object detection framework that formulates detection as a direct regression problem, enabling efficient inference while achieving high detection performance with relatively low computational cost and model complexity [37]. In this framework, a feature map is represented as $F\in R^{C\times H\times W}$, where *C*, *H*, and *W* denote the number of channels, height, and width, respectively.

The bounding box regression branch is optimized using a combination of Complete Intersection over Union (CIoU) loss and Distribution Focal Loss (DFL) to improve localization accuracy. In addition, ${\mathrm{loss}}_{\mathrm{cls}}$, ${\mathrm{loss}}_{\mathrm{box}}$, and ${\mathrm{loss}}_{\mathrm{dfl}}$ all contribute to the ${\mathrm{loss}}_{\mathrm{total}}$, affecting both classification and localization accuracy. Non-maximum suppression is subsequently applied to remove redundant predictions and generate the final detection results.

Despite its efficient detection capability, the baseline YOLO architecture still relies on static feature aggregation mechanisms, which motivate the introduction of the proposed CAEFRER framework described in the following section.

### 3.2. Architectural Overview of the CAEFRER-YOLO11

**Figure 2:**
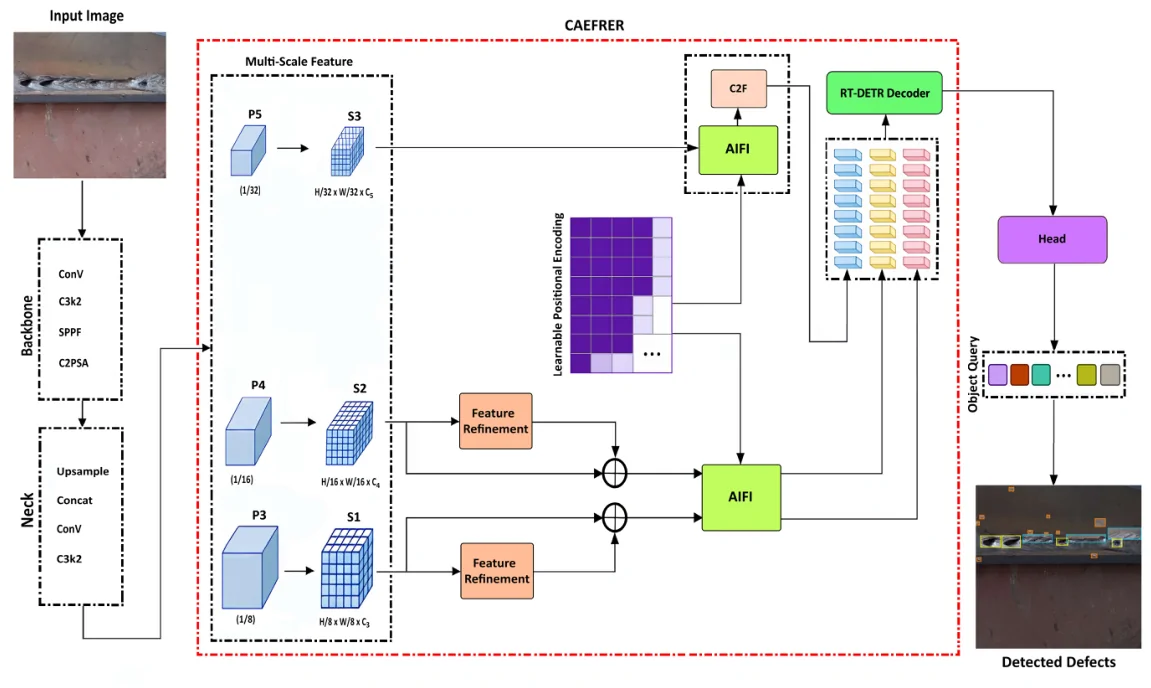
Overall architecture of the proposed CAEFRER-YOLO11 model for weld defect detection.

The proposed hybrid CAEFRER-YOLO11 architecture is illustrated in Figure 2 and consists of four main components: a YOLO11 backbone based on a CSP-inspired design for feature extraction, a PAN-FPN-based neck for multi-scale feature aggregation, the proposed CAEFRER framework positioned between the neck and the detection head, incorporating an RT-DETR-based decoder, and a separate detection head. The CAEFRER-YOLO11 architecture combines YOLO11-based multi-scale feature extraction and refinement with transformer-based object detection, enabling end-to-end weld defect detection. Following multi-scale feature extraction at the P3, P4, and P5 levels, the resulting feature maps are processed independently through three feature refinement pathways while preserving their respective spatial resolutions.

In the P3 and P4 branches, RepVGG-based feature refinement enhances local feature representations. Element-wise addition is performed independently within each of these branches to combine the refined features with their corresponding original feature maps, thereby establishing residual connections. These residual connections preserve the original P3 and P4 feature information while incorporating locally refined representations, without altering their spatial dimensions [38]. Learnable positional encoding (LPE) is applied to the resulting P3 and P4 feature maps before AIFI processing to preserve spatial information and support the modeling of spatial relationships within the feature representations. The resulting feature maps are subsequently processed by their respective AIFI modules to capture long-range dependencies and contextual interactions, thereby improving feature discrimination for weld defect detection [39]. In the P5 branch, LPE is applied to the feature map before AIFI processing to enhance contextual feature representations, followed by C2f (Cross Stage Partial with two convolutions)-based feature transformation to strengthen local feature aggregation and information flow. The three independently refined feature maps retain their respective spatial resolutions and are subsequently provided to the RT-DETR-based decoder for multi-scale object-level feature processing.

The refined multi-scale feature maps are subsequently processed by the RT-DETR-based decoder integrated within the proposed CAEFRER framework, enabling transformer-based, end-to-end, anchor-free object detection [40]. The decoder utilizes object queries automatically selected from encoder features to refine object-level representations. Finally, the decoder outputs are passed to the separate detection head for defect classification and bounding-box localization, producing the final weld defect detection results.

In addition, the integration of RepVGG-style re-parameterizable blocks into the feature refinement stage enables the model to capture defect patterns with diverse spatial scales and morphological characteristics. During training, the multi-branch structure learns complementary representations, whereas during inference it is structurally re-parameterized into an equivalent single-path convolution.

### 3.3. Proposed CAEFRER Framework

YOLO11 baselines exhibit three main failure modes in industrial weld defect detection: limited sensitivity to small defects due to weak fine-scale feature responses, false positive activations caused by background artifacts such as spatter and reflections, and unstable feature representations under noisy and low-contrast imaging conditions. These limitations indicate the need for a reliability-aware feature refinement strategy. To address these challenges, the proposed CAEFRER framework explicitly associates each functional component with a specific failure mode. The AIFI modules employ multi-head self-attention to capture long-range dependencies and contextual relationships within individual feature scales, thereby enhancing feature discrimination and sensitivity to small weld defects [41].

The attention operation within AIFI employs a similarity-driven weighting mechanism to adaptively emphasize relevant spatial features within each feature scale [42]. This attention-based weighting can reduce the influence of less informative responses and emphasize defect-sensitive regions, thereby supporting more discriminative feature representations for subsequent detection.

**Figure 3:**
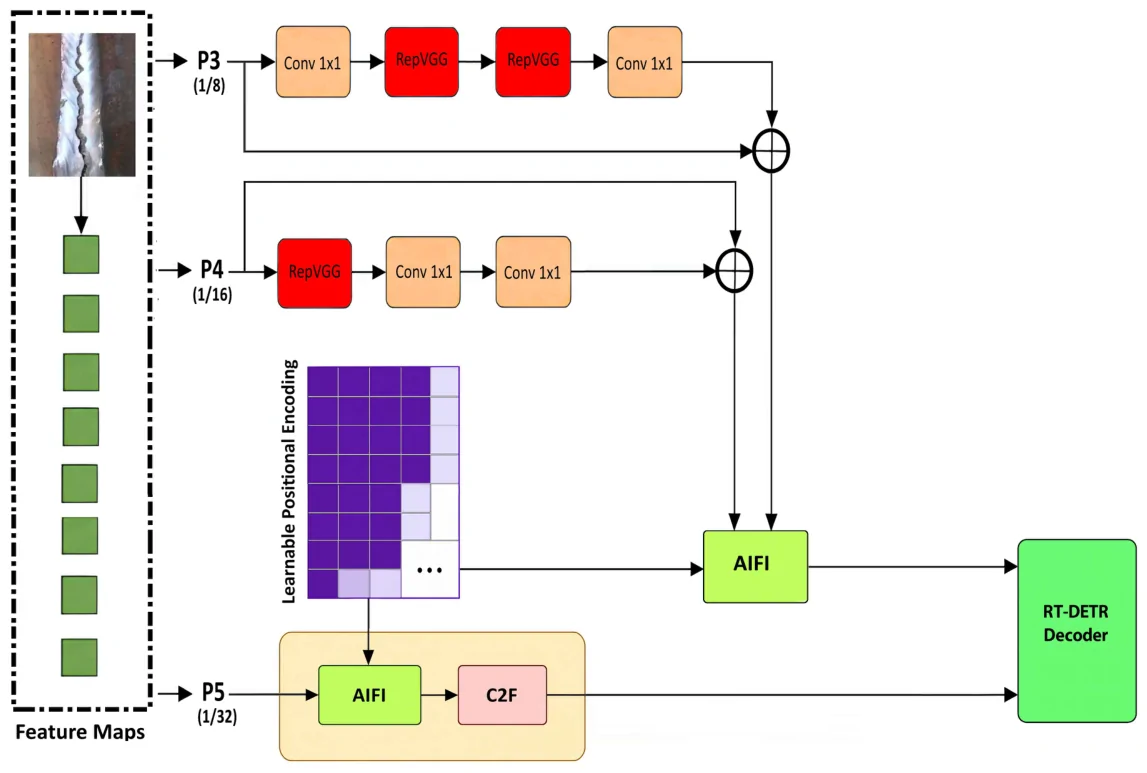
CAEFRER framework: three parallel branches for multi-scale feature refinement and contextual modeling, with branch-wise residual addition to enhance small-object detection through a hybrid architecture.

Figure 3 shows the CAEFRER framework, which employs three parallel feature enhancement branches with independent feature refinement and contextual modeling. Multi-scale features from the backbone and neck are processed in parallel to capture complementary local, contextual, and structural information while preserving their respective spatial resolutions.

In the first branch, the input feature map $F_{P3}$ is processed through a 1×1 convolution (Conv), followed by two sequential RepVGG blocks optimized with grouped convolutions (g=2), and a final 1×1 convolution to extract fine-grained local features and structural details, yielding the feature representation $F_{\mathrm{small}}$:

(1) $$F_{\mathrm{small}}={\mathrm{Conv}}_{1\times 1}\left(\mathrm{RepVGG}\left(\mathrm{RepVGG}\left({\mathrm{Conv}}_{1\times 1}\left(F_{P3}\right)\right)\right)\right)$$

In the second branch, the input feature map $F_{P4}$ is processed through a RepVGG-B0 block followed by two sequential 1×1 Conv blocks, where the RepVGG-B0 block provides an alternative to conventional 3×3 ConVs [43]. This design enhances local feature representations while capturing defect patterns with diverse morphological characteristics, yielding the feature representation $F_{\mathrm{medium}}$:

(2) $$F_{\mathrm{medium}}={\mathrm{Conv}}_{1\times 1}\left({\mathrm{Conv}}_{1\times 1}\left(\mathrm{RepVGG}(F_{P4})\right)\right)$$

Here, the nested operations denote the sequential application of the convolutional layers. Subsequently, element-wise addition is performed independently within the P3 and P4 branches, combining $F_{\mathrm{small}}$ and $F_{\mathrm{medium}}$ with their corresponding original feature maps, respectively, to establish residual connections while preserving their spatial dimensions.

(3) $$F_{P3}^{\mathrm{res}}=F_{P3}⊕F_{\mathrm{small}}$$

(4) $$F_{P4}^{\mathrm{res}}=F_{P4}⊕F_{\mathrm{medium}}$$

In the third branch, $F_{P5}$ is then fed into the AIFI module. This module consists of multi-head self-attention, FFN, and Layer Normalization layers. In addition, LPE is independently applied to preserve spatial information. During attention computation, the similarity matrix $A_{ij}$, representing correlations between query and key embeddings, is used to adaptively weight feature interactions prior to aggregation. The scaled attention score $A_{ij}$ is mathematically formulated as follows:

(5) $$A_{ij}=\frac{Q_{i}K_{j}^{T}}{\sqrt{d_{K}}}$$

where $Q_{i}$ is the query vector at position *i*, $K_{j}$ is the key vector at position *j*, *T* denotes the transpose operation, and $d_{K}$ is the dimensionality of the key vectors. This processing pathway adaptively models spatial relationships and contextual dependencies, producing the refined feature map $F_{P5}^{\mathrm{enc}}$:

(6) $$F_{P5}^{\mathrm{enc}}=C2f\left(\mathrm{AIFI}(F_{P5})\right)$$

Subsequently, the P3 and P4 residual feature maps are independently processed by AIFI, as follows:

(7) $$F_{P3}^{\mathrm{enc}}=\mathrm{AIFI}\left(F_{P3}^{\mathrm{res}}\right)$$

(8) $$F_{P4}^{\mathrm{enc}}=\mathrm{AIFI}\left(F_{P4}^{\mathrm{res}}\right)$$

Finally, the refined feature maps $F_{P3}^{\mathrm{enc}}$, $F_{P4}^{\mathrm{enc}}$, and $F_{P5}^{\mathrm{enc}}$ remain independent, preserving their respective spatial resolutions while their channel dimensions are aligned before being forwarded to the RT-DETR-based decoder for multi-scale object-level feature processing. The resulting decoder output $F_{\mathrm{out}}$ is mathematically expressed as follows:

(9) $$F_{\mathrm{out}}=\mathrm{RT}\text{-}\mathrm{DETRDecoder}\left(F_{P3}^{\mathrm{enc}},F_{P4}^{\mathrm{enc}},F_{P5}^{\mathrm{enc}}\right)$$

Unlike the original CAEFRE-DETR architecture, which employs multiple Deformable DETR, Multi-Layer Perceptron, and 3×3 Conv blocks for contextual interaction, the proposed CAEFRER framework integrates AIFI and C2f modules with RepVGG-based structural re-parameterization [44]. This framework improves compatibility with YOLO11 while preserving effective contextual feature interactions.

### 3.4. Architectural Overview of the RT-DETR

In RT-DETR, the encoded features are processed by a transformer-based decoder, which refines object queries through cross-attention and directly predicts class labels and bounding box coordinates in an end-to-end manner. In addition, ${\mathrm{loss}}_{l1}$, ${\mathrm{loss}}_{\mathrm{giou}}$, and ${\mathrm{loss}}_{\mathrm{cls}}$ together form the ${\mathrm{loss}}_{\mathrm{total}}$, affecting both classification and localization accuracy. RT-DETR variants employ different backbones, such as HGNetv2 and ResNet, which affect the trade-off between feature representation, computational complexity, and detection performance. In this study, the Ultralytics RT-DETR with an HGNetv2 backbone was selected as a baseline for comparison with the proposed model under identical evaluation conditions.

### 3.5. Datasets and Evaluation Metrics

Two datasets were used in this study: the WDD and the proposed HWD datasets. The WDD dataset contains 1437 RGB images, comprising 1152 training images, 210 validation images, and 75 test images, with an approximate train:validation:test split ratio of 15.4:2.8:1. The WDD dataset was obtained from the “Weld Defect Detection Dataset” published by Sukma Adhi Wijaya on the Kaggle platform [45]. This dataset comprises five defect categories: Adjacent Defects (Class 0), Integrity Defects (Class 1), Geometry Defects (Class 2), Post-Processing Defects (Class 3), and Non-Fulfillment Defects (Class 4). To ensure consistency with the proposed three-class classification scheme, Post-Processing Defects (Class 3) were merged with Adjacent Defects (Class 0) to form Surface Defects, while Non-Fulfillment Defects (Class 4) were merged with Integrity Defects (Class 1) to form Structural Defects. Geometry Defects (Class 2) remained unchanged.

The proposed HWD dataset consists of 2073 original RGB images collected from real-world welding processes. The experimental settings and imaging details of the self-collected HWD dataset are summarized in Table 1. By combining the public WDD dataset with our dataset, a hybrid dataset comprising 3510 RGB images was constructed for model training and evaluation. The proposed dataset was divided into training (2784 images, 79.32%), validation (537 images, 15.30%), and test (189 images, 5.38%) subsets, corresponding to an approximate train:validation:test ratio of 14.7:2.8:1.

**Figure 4:**
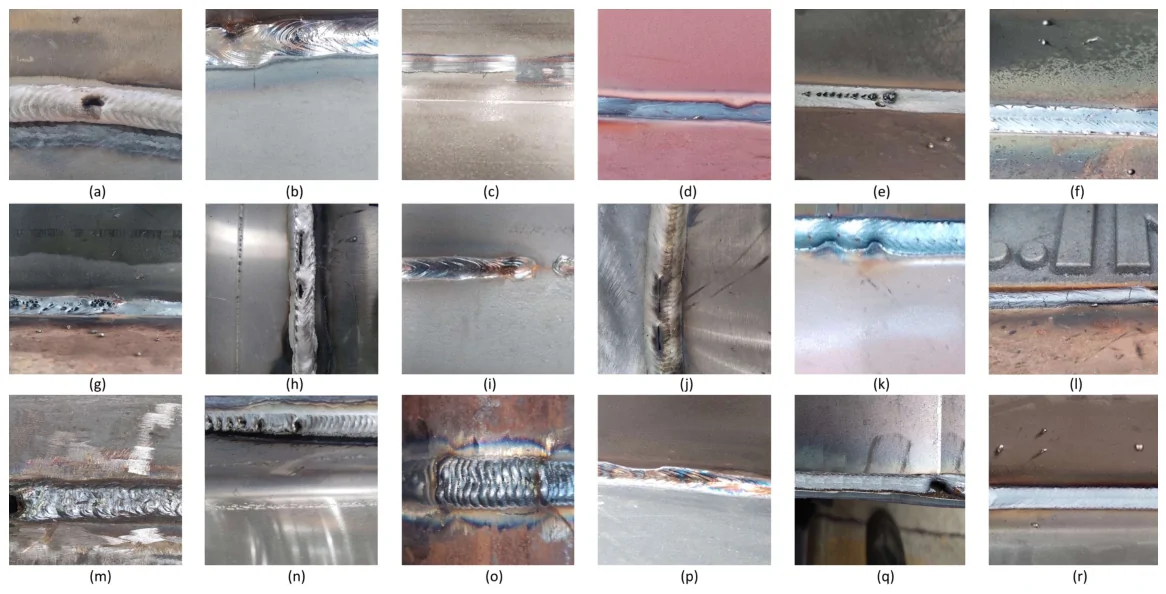
Representative samples from the proposed HWD: (**a**,**m**,**n**) burn-through; (**b**,**o**) irregular bead profiles; (**c**,**l**) microcracks; (**e**,**g**) porosity; (**f**,**r**) spatter; (**d**,**h**,**j**,**k**,**p**) inadequate weld leg; and (**i**,**q**) skipped weld.

Our proposed hybrid dataset is organized into three defect categories: surface (Class 0) with 3425 instances, structural (Class 1) with 2073 instances, and geometric (Class 2) with 2062 instances, resulting in a total of 7560 defect instances. In contrast, the WDD dataset contains 3972 defect instances, comprising 1879 surface (Class 0, 47.30%), 1029 structural (Class 1, 25.91%), and 1064 geometric (Class 2, 26.79%) instances. Specifically, the welding defects in the HWD dataset were categorized based on their location relative to the weld region and their distinct geometric characteristics. Class 0 (Surface Defects) comprises superficial defects occurring around the weld region, including spatter, arc strikes, burrs, slag deposits outside the weld region, and scratches. Class 1 (Structural Defects) includes defects that directly compromise weld integrity and structural continuity, such as crater cracks, microcracks, internal slag and inclusions, porosity, burn-through, and skipped weld and inadequate weld leg corresponding to 35–69% of the weld region. Class 2 (Geometry Defects) comprises defects that primarily affect the geometry and profile of the weld bead, including undercut, overlap, surface irregularities, excessive weld metal, concavity, irregular bead profiles, and inadequate weld leg, corresponding to 5–34% of the weld region. Representative samples of the dataset are presented in Figure 4. To enhance material diversity and improve model robustness, the dataset includes weld images of both steel and aluminum materials.

All images were manually annotated using the Makesense.ai platform with object-level bounding boxes corresponding to the three defect categories. The annotations were converted into formats compatible with both YOLO-based and RT-DETR-based detectors. The performance of the object detection models was evaluated using standard detection metrics. To ensure the accuracy and reliability of the dataset, the ground-truth annotations and defect classifications were reviewed and validated by a certified International Welding Engineer (IWE).

Precision and recall are widely used to evaluate modern object detection models, including YOLO- and RT-DETR-based detectors. Precision measures the proportion of correct predictions among all predicted positives using TP and FP, whereas recall measures the proportion of actual objects correctly detected, accounting for FN [46].

(10) $$\mathrm{Precision}=\frac{\mathrm{TP}}{\mathrm{TP}+\mathrm{FP}}$$

(11) $$\mathrm{Recall}=\frac{\mathrm{TP}}{\mathrm{TP}+\mathrm{FN}}$$

The mAP is reported at Intersection over Union (IoU) thresholds of 0.5 (${\mathrm{mAP}}_{50}$) and 0.5–0.95 (${\mathrm{mAP}}_{50-95}$), enabling assessment of both overall detection performance and localization accuracy under increasingly strict conditions. Moreover, scale-aware performance is evaluated using object-size-specific metrics, namely ${\mathrm{mAP}}_{s}$, ${\mathrm{mAP}}_{m}$, and ${\mathrm{mAP}}_{l}$, corresponding to small, medium, and large objects, respectively. These metrics provide a more detailed assessment of the model’s robustness across different object scales, which is particularly important in small defect detection tasks. Scale-specific performance was evaluated using ${\mathrm{mAP}}_{s}$, ${\mathrm{mAP}}_{m}$, and ${\mathrm{mAP}}_{l}$ for small (<$32^{2}$ pixels), medium ($32^{2}$–$96^{2}$ pixels), and large (>$96^{2}$ pixels) objects, respectively.

In addition, the dataset exhibits significant variability in object scale and visibility conditions. Regarding size distribution, 36.30% of the annotated defects are classified as small, 44.25% as medium-sized, and 19.45% as large, highlighting the challenges of multi-scale object detection.

### 3.6. CAM-Based Model Interpretability Methods

A qualitative-to-quantitative consistency analysis was conducted to evaluate the interpretability of the proposed CAEFRER framework. Compared to our YOLO11-based model, the baselines exhibit more scattered activation patterns and a weaker focus on small (<32×32 pixels) defect bounding boxes. To quantitatively evaluate the faithfulness of these activation maps, the Average Drop (AD) metric was employed using a region-deletion strategy. In the M1 condition, high-activation regions identified by Eigen-CAM were masked, whereas M2 involved masking background regions. A higher AD under M1 indicates greater sensitivity to the removal of high-activation regions, while an AD closer to zero under M2 indicates lower sensitivity to background masking. Furthermore, a negative AD value indicates an increase in model confidence following the removal of background regions. The notation used for the XAI metrics is summarized in Table 2.

(12) $$\mathrm{AD}=\frac{1}{N}\sum_{i=1}^{N}\left(\frac{\;Y_{i}^{c}-O_{i}^{c})}{Y_{i}^{c}}\right)\times 100,$$

To further evaluate the consistency of Eigen-CAM explanations, the Standard Deviation of Decrease (SDD) is calculated. This metric measures the variability of the confidence reductions across all test samples. A lower SDD indicates more stable and consistent attention behavior, whereas a higher value suggests greater variability in the explanatory regions.

(13) $$\mathrm{SDD}=\sqrt{\frac{1}{N}\sum_{i=1}^{N}{\left(x_{i}-\mu \right)}^{2}}$$

The Eigen-CAM-based analysis was used as an auxiliary validation approach to examine the consistency of model attention, with AD and SDD used for quantitative evaluation, rather than as a standalone contribution of this study. In this method, PCA is applied to activation feature maps to extract principal components that capture the dominant spatial patterns, which are subsequently used to generate class activation maps without requiring gradient backpropagation [47]. In the selected explainability method, the first principal component, which captures the maximum variance in the feature maps, is used to generate the activation heatmap without relying on gradient computation.

## 4. Results and Discussion

### 4.1. Experimental Setup

In this study, all models were trained for 100 epochs using AdamW (lr = 0.0005, momentum = 0.937, weight decay = 0.00008), with 640 × 640 inputs and batch size 16. The computational environment and software specifications are summarized in Table 3.

All baseline models were initialized with their officially released pretrained weights and trained using their original architectures without modifications. The proposed CAEFRER-YOLO11 model was initialized with pretrained YOLO11 weights and integrated the proposed feature refinement framework and an RT-DETR-based decoder. All models were trained and evaluated under the same experimental settings to ensure a consistent and fair comparison.

The comparative analysis of eight models in terms of parameters, inference time (IT), and FLOPs, including the proposed model, is presented in Table 4 on the HWD dataset. The IT for each model was calculated as the average per-image execution time across the 189 test images, including both pre-processing and post-processing times, using a batch size of 1 with TensorRT FP32 acceleration. The reported IT excludes external end-to-end latency components, such as image capture, network transmission, and output/display processing. The CAEFRER framework adds approximately 9.20 M parameters to the YOLO11-L baseline, distributed across three branches: ∼1.65 M, ∼3.81 M, and ∼3.74 M, respectively. Additionally, the RT-DETR-based decoder and associated detection components account for ∼4.50 M parameters. By capturing high-level semantic information and global contextual cues, these modules are designed to improve feature representation and localization of small welding defects, such as microcracks and micropores, which are often characterized by low contrast and limited spatial extent.

After training on the same domain-specific dataset with identical hyperparameter setting, all models are evaluated using standard object detection metrics, and the comparative results, including ablation studies and qualitative analyses, are presented in the subsequent section.

### 4.2. Comparison with Other Algorithms and Discussions

**Figure 5:**
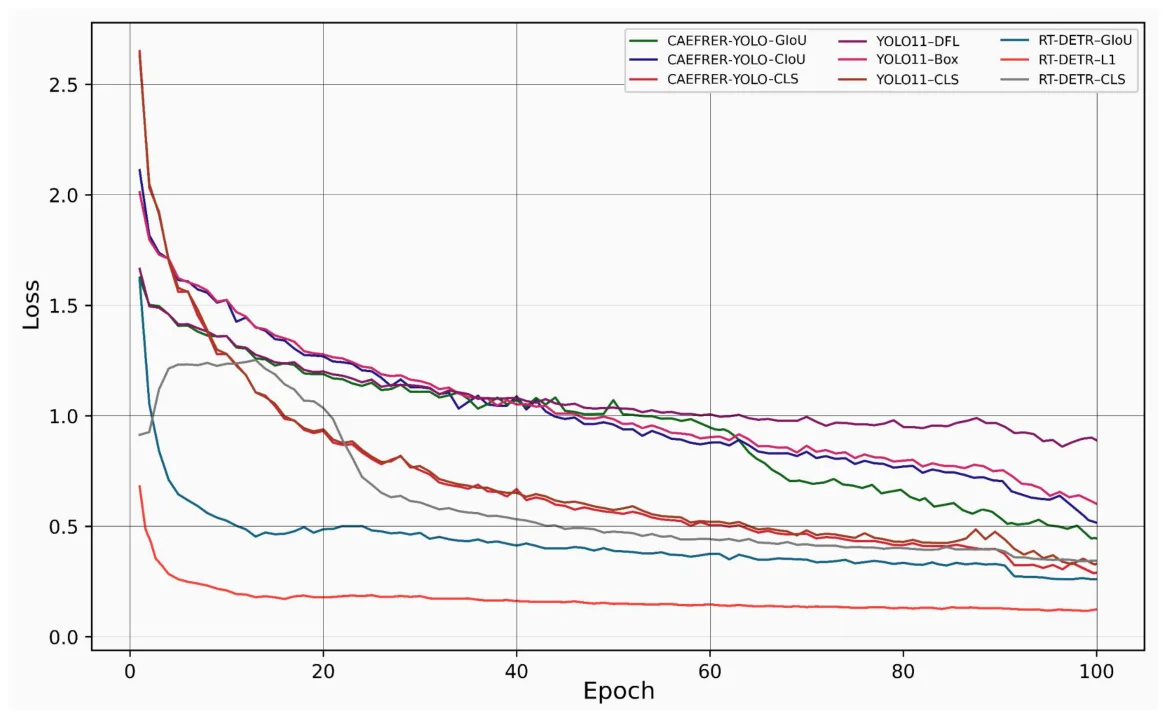
Training loss convergence of CAEFRER-YOLO11, YOLO11-L, and RT-DETR-L.

To comprehensively evaluate the proposed framework against existing models, comparative experiments were conducted on the HWD dataset using three object detection models, including the proposed CAEFRER-YOLO11, under consistent training configurations. Figure 5 illustrates the training loss curves, showing that the YOLO-based models exhibit substantial reductions in their training losses during the first 20 epochs, followed by gradual convergence after approximately 53 epochs.

This figure compares the training loss convergence of CAEFRER-YOLO11, YOLO11-L, and RT-DETR-L over 100 epochs. The CIoU-based ${\mathrm{loss}}_{\mathrm{box}}$ and ${\mathrm{loss}}_{\mathrm{giou}}$ of CAEFRER-YOLO11 exhibit similar convergence trends to the corresponding ${\mathrm{loss}}_{\mathrm{box}}$ of YOLO11-L and ${\mathrm{loss}}_{\mathrm{giou}}$ of RT-DETR-L, respectively. Classification losses are also compared across all three models. CAEFRER-YOLO11 achieves a final ${\mathrm{loss}}_{\mathrm{cls}}$ of 0.279, representing reductions of 11.99% and 15.20% compared with YOLO11-L (0.317) and RT-DETR-L (0.329), respectively. Overall, the loss curves exhibit decreasing trends throughout training, indicating progressive convergence, although the different regression loss formulations are not directly equivalent in magnitude.

To evaluate CAEFRER-YOLO11, comparative experiments were conducted with representative YOLO11 variants and RT-DETR under identical settings on the HWD dataset. The comparative results are summarized in Table 5. Each model was trained with three random seeds (25, 30, and 42). Performance was assessed using standard object detection metrics, including ${\mathrm{mAP}}_{50–95}$, ${\mathrm{mAP}}_{s}$, ${\mathrm{mAP}}_{m}$, ${\mathrm{mAP}}_{l}$, and ${\mathrm{mAR}}_{100}$.

As presented in Table 5, the proposed CAEFRER-YOLO11 achieved the highest ${\mathrm{mAP}}_{50–95}$ among the compared models, with a mean value of 55.73±1.58%. The best performance was achieved at epoch 99. This outperformed RT-DETR-L (54.98% ± 1.47% at epoch 83) and YOLO11-L (53.64% ± 1.84% at epoch 95). In addition to achieving the highest ${\mathrm{mAP}}_{50–95}$, ${\mathrm{mAP}}_{s}$, and ${\mathrm{mAP}}_{m}$ values among the evaluated models, the proposed model demonstrates superior overall detection capability, particularly for small and medium-sized defects. Although the RT-DETR-L variant achieves higher ${\mathrm{mAR}}_{100}$ and ${\mathrm{mAP}}_{l}$ values, CAEFRER-YOLO11 provides more balanced overall detection performance with improved feature representation and scale-aware detection capability.

Table 6 presents the class-wise performance of the evaluated methods across two dataset sizes. For Class 0, the proposed method consistently outperformed YOLO11-L and RT-DETR-L in precision and ${\mathrm{AP}}_{50}$. On WDD, it achieved the highest precision and ${\mathrm{AP}}_{50}$, with gains of 2.67/3.61 pp and 6.25/4.56 pp over YOLO11-L/RT-DETR-L, respectively, while RT-DETR-L achieved the highest recall. On HWD, the proposed method again achieved the highest precision, ${\mathrm{AP}}_{50}$, and recall, with gains of 1.27/2.60 pp, 5.37/3.89 pp, and 2.74/0.81 pp, respectively.

For Class 1, the proposed method achieved the highest precision on the WDD dataset, exceeding YOLO11-L and RT-DETR-L by 1.95 and 1.28 pp, respectively. On the HWD dataset, RT-DETR-L led in precision, recall, and ${\mathrm{mAP}}_{50}$, while the proposed method exceeded YOLO11-L in recall by 0.74 pp.

For Class 2, RT-DETR-L led in precision and ${\mathrm{mAP}}_{50}$ on WDD, whereas the proposed method achieved the highest recall, exceeding YOLO11-L and RT-DETR-L by 2.47 and 0.45 pp. On HWD, the proposed method achieved the highest precision and ${\mathrm{mAP}}_{50}$, outperforming RT-DETR-L by 1.51 and 0.36 pp, respectively.

**Figure 6:**
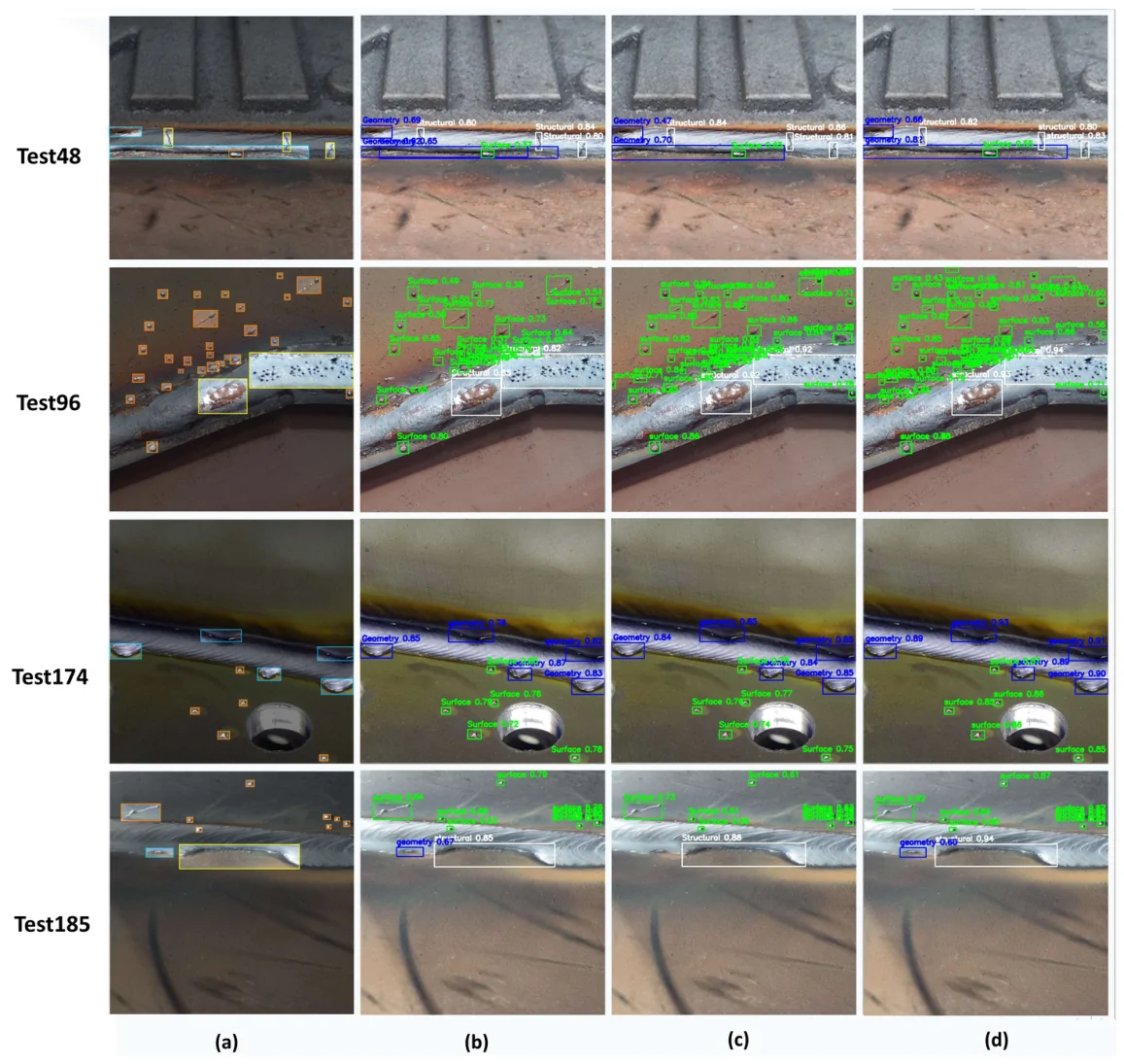
Qualitative comparison of the detection results on Test48, Test96, Test174, and Test185: (**a**) ground truth, (**b**) YOLO11-L, (**c**) RT-DETR-L, and (**d**) the proposed method predictions.

Figure 6 qualitatively compares YOLO11, RT-DETR, and the proposed method on Test48, Test96, Test174, and Test185 (seed 42). While all models detect the main weld regions, differences are observed in localization consistency and false-positive suppression. In Test96, containing numerous small, closely distributed surface defects, YOLO11 and RT-DETR generate dense predictions, whereas the proposed method provides more consistent detections with less clutter. In Test174, the proposed method achieves tighter bounding-box alignment with the weld geometry while retaining relevant surface-defect detections. In Test185, it provides more confident and coherent localization of the long, low-contrast structural defect, demonstrating improved robustness for weak-contrast weld structures.

Comparative evaluations indicate that CAEFRER-YOLO11 provides a favorable balance between detection performance and localization consistency, particularly for small and medium-sized defects. However, RT-DETR variants achieve higher recall for some categories and better performance on large objects, indicating limitations in larger or more diverse defect regions.

These findings indicate that CAEFRER-YOLO11 exhibits certain limitations, particularly in detecting structural defects (Class 1). This suggests that the model’s emphasis on fine-scale feature refinement may provide comparatively limited benefits for detecting large-scale defects. Furthermore, inference results for structural defects in the HWD dataset reveal missed detections and low-confidence predictions compared with RT-DETR-L. In particular, cases were observed in which two or more distinct porosity or burn-through defects were detected as a single defect. These findings highlight the limitations of the proposed model in detecting and distinguishing certain structural defects and are supported by observations obtained from inference on test images.

Additionally, although the HWD dataset includes weld images of ST37 carbon steel, Hardox 450 steel, and aluminum produced using MAG, MIG, and SAW processes, its applicability to other metals, alloys, and welding processes, such as underwater welding, has not yet been experimentally validated. Nevertheless, the proposed model may also be applicable to micro-jet cooling-assisted welding applications due to its compatibility with conventional gas-shielded welding processes; however, further experimental studies are required to validate this potential applicability. Furthermore, the HWD dataset does not include dissimilar material joints; therefore, the applicability of CAEFRER-YOLO11 to dissimilar welding has not yet been experimentally validated.

Qualitative results further show reduced redundant detections and improved localization consistency for closely distributed and low-contrast defects. Nevertheless, performance remains dependent on defect scale and visual characteristics, and validation on independent welding datasets is needed to confirm generalization. Overall, the results suggest that the main contribution of CAEFRER-YOLO11 is not simply achieving the highest score across all metrics, but providing a more balanced detection performance, particularly for challenging small and low-contrast weld defects.

### 4.3. Further Experimental Analysis of CAEFRER-YOLO11

In this study, additional experiments were performed using 10-fold cross-validation on the HWD dataset. All experiments and subsequent evaluations were conducted under the same hyperparameter settings as those used in the main experiments. This evaluation aimed to assess the robustness, stability, and generalization capability of the proposed framework.

**Figure 7:**
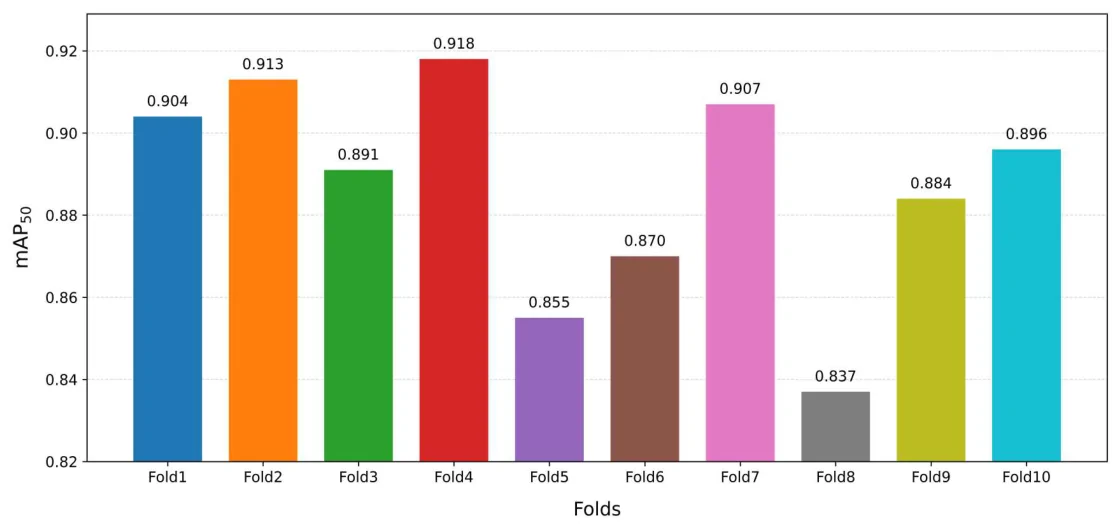
10-fold CV results for ${\mathrm{mAP}}_{50}$ using the proposed model with a fixed random seed of 42.

On the HWD dataset, with a random seed of 42, 10-fold cross-validation (CV) was performed using a 9:1 split, corresponding to approximately 2989 training images (90%) and 332 validation images (10%) per fold, thereby ensuring a uniform distribution across all folds. Figure 7 presents the 10-fold cross-validation results for ${\mathrm{mAP}}_{50}$ of the proposed CAEFRER-YOLO11 model. The model achieved a mean ${\mathrm{mAP}}_{50}$ of 0.8875±0.0263, with a 95% confidence interval (CI) of [0.8687,0.9063]. The fold-specific ${\mathrm{mAP}}_{50}$ values ranged from 0.837 to 0.918, with the highest value obtained in Fold 4 (0.918) and the lowest in Fold 8 (0.837). The coefficient of variation was 2.97%, indicating relatively low variability across the folds.

**Figure 8:**
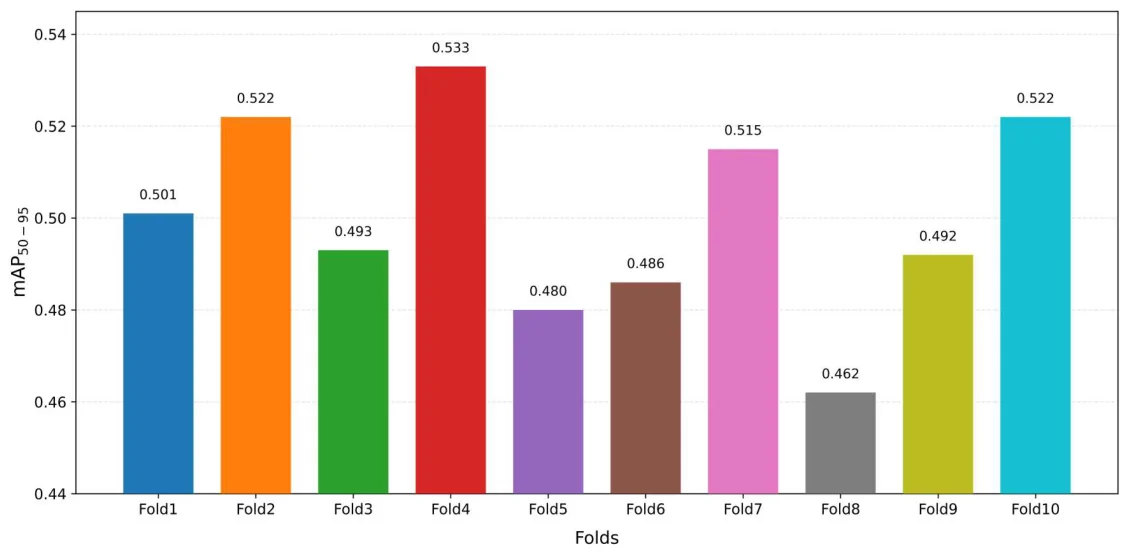
10-fold CV results for ${\mathrm{mAP}}_{50–95}$ using the proposed model with a fixed random seed of 42.

Figure 8 shows the 10-fold cross-validation results for ${\mathrm{mAP}}_{50–95}$ using the proposed CAEFRER-YOLO11 model. The model obtained a mean ${\mathrm{mAP}}_{50–95}$ of 0.5006±0.0222, with a 95% confidence interval (CI) of [0.4847,0.5165]. The fold-specific values ranged from 0.462 to 0.533, with Fold 4 achieving the highest value (0.533) and Fold 8 the lowest (0.462). The coefficient of variation was 4.43%, indicating slightly greater relative variability than that observed for ${\mathrm{mAP}}_{50}$. The 10-fold cross-validation results for both ${\mathrm{mAP}}_{50}$ and ${\mathrm{mAP}}_{50–95}$ are summarized in Table 7.

Table 8 presents the ablation study and baseline comparison across three random seeds. CAEFRER-YOLO11 outperformed YOLO11-L and RT-DETR-L by 3.18 and 1.31 pp, respectively. Compared with YOLO11-L, the P3-only, P4-only, and combined P3–P4 enhancements improved ${\mathrm{mAP}}_{50}$ by 1.75, 0.96, and 2.89 pp, respectively. The additional P5 enhancement further increased ${\mathrm{mAP}}_{50}$ by 0.29 pp, demonstrating the effectiveness of the complete architecture.

### 4.4. Visualization Results and Discussions

To further investigate the spatial focus and interpretability of the evaluated models, Eigen-CAM activation maps were generated and analyzed on the HWD dataset for models trained using three different seeds (25, 30, and 42). The heatmaps qualitatively assess attention to discriminative weld and defect regions while avoiding irrelevant backgrounds. Quantitative analyses of high-activation (M1) and background (M2) regions further support these observations.

**Figure 9:**
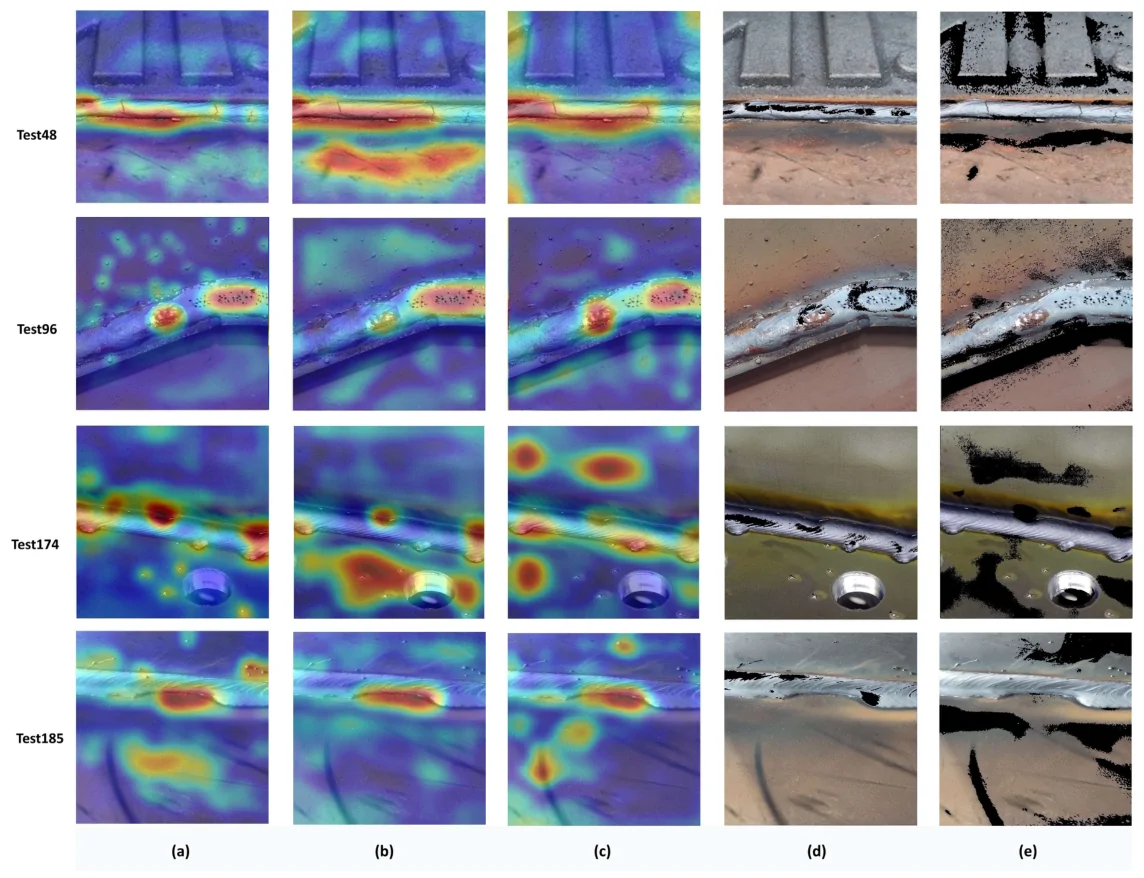
Eigen-CAM heatmaps and corresponding region masks for Test48, Test96, Test174, and Test185: (**a**) CAEFRER-YOLO11, (**b**) YOLO11-L, (**c**) RT-DETR-L, (**d**) M1 masks, and (**e**) M2 masks.

Figure 9 presents the Eigen-CAM activation maps obtained from the proposed method, YOLO11, and RT-DETR for Test48, Test96, Test174, and Test185. The proposed method generally produces more concentrated activation responses around the weld seam and defect regions, whereas YOLO11 and RT-DETR exhibit broader activations extending into surrounding background areas.

In Test48, the proposed method concentrates the strongest activations along the weld seam, providing a clearer correspondence with the actual weld structure. For Test96, it produces distinct activation peaks around the visible defect regions, while YOLO11 and RT-DETR show more dispersed responses and additional activation in surrounding areas. In Test174 and Test185, the proposed method maintains more localized responses along the relevant weld regions, whereas the baseline models exhibit broader high-activation areas extending into non-relevant background regions.

Across the four evaluated samples(Test48, Test96, Test174, and Test185), the proposed method achieved an AD of 11.95% and an SDD of 5.72% for M1, whereas M2 yielded an AD of −3.52% and an SDD of 4.87%. In the perturbation analysis, 20% of the background regions was consistently masked for M2 across all test images, while the masked high-activation regions in M1 represented 17.1–22.8% of the corresponding high-activation areas, equivalent to approximately 2.5% of the total image area.

As shown in Table 9, CAEFRER-YOLO11 achieved the highest AD of 9.23% for M1 and the lowest AD of −1.63% for M2, indicating a stronger response to high-activation regions and limited sensitivity to background regions. These findings provide quantitative support for the effectiveness of the proposed feature-refinement strategy in emphasizing discriminative weld-defect features while suppressing irrelevant background responses.

AD and SDD metrics provide quantitative evidence of sensitivity to relevant and background regions, while overlap-based metrics such as Attention-Region Precision (APR) could further assess the spatial correspondence between activation maps and annotated defect regions. Incorporating APR and related spatial metrics in future studies could provide a more comprehensive evaluation of XAI localization fidelity.

## 5. Conclusions

This study demonstrated that, compared with the baseline models, the proposed CAEFRER framework improved the evaluation metrics for weld defect detection, particularly for small-sized defects that are more challenging to detect. Furthermore, the findings obtained on the HWD dataset indicate that the additional data incorporated into the existing WDD dataset contributed to reducing the performance gap in the structural defect class, which had previously exhibited comparatively lower detection performance, thereby supporting a more balanced class-wise detection performance.

Under the same evaluation conditions, the proposed model achieved relative improvements of 3.90% and 1.36% in ${\mathrm{mAP}}_{50–95}$ compared with YOLO11-L and RT-DETR-L, respectively. In particular, ${\mathrm{mAP}}_{s}$ showed relative improvements of 9.76% and 6.05%, respectively, for small objects with bounding-box areas below $32^{2}$ pixels. For the class-wise analysis on WDD, surface defects showed relative ${\mathrm{mAP}}_{50}$ gains of 8.32% and 5.94% over YOLO11-L and RT-DETR-L, respectively. Overall, CAEFRER-YOLO11 achieved the highest ${\mathrm{mAP}}_{50–95}$ among the evaluated models. On HWD, it also achieved relative ${\mathrm{mAP}}_{50}$ gains of 6.24% and 4.44% for surface defects compared with YOLO11-L and RT-DETR-L, respectively. Small weld defects, including microcracks, micropores, burn-through, and spatter, were identified. Based on established welding principles, these defects may be associated with factors such as excessive heat input, joint gap variations, shielding gas entrapment, unstable metal transfer, and surface contamination. However, since the welding parameters were not systematically varied to establish cause-and-effect relationships, the underlying causes of these defects could not be experimentally confirmed in this study.

The 10-fold CV results further demonstrated stable performance of CAEFRER-YOLO11 across both evaluation metrics. For ${\mathrm{mAP}}_{50}$, the model achieved a mean of 0.8875 with a standard deviation of 0.0263, with fold-specific values ranging from 0.837 to 0.918. For ${\mathrm{mAP}}_{50–95}$, the model achieved a mean of 0.5006 with a standard deviation of 0.0222, with fold-specific values ranging from 0.462 to 0.533.

The XAI analysis further supported the spatial focus of CAEFRER-YOLO11. Under M1 masking, the proposed model showed stronger activation sensitivity than YOLO11-L and RT-DETR-L, while M2 masking resulted in limited changes in its activation response. Overall, these results indicate that CAEFRER-YOLO11 produces more focused and discriminative activation patterns than the baseline models.

Future work will focus on practical deployment, evaluating the applicability of CAEFRER-YOLO11 to different welding materials, and assessing real-time performance under actual industrial operating conditions, including deployment on resource-constrained platforms such as NVIDIA Jetson Nano with model and hardware optimization. End-to-end latency, including capture, preprocessing, inference, post-processing, network transmission, and output generation, will also be investigated. For explainability, Pointing Game Accuracy and APR will be incorporated, together with comparative evaluation using CAM methods such as Dynamic Thresholding Grad-CAM and Grad-CAM++, to assess spatial fidelity and explanation robustness.

## Figures and Tables

**Table 1:** Welding parameters and image acquisition conditions for the self-collected dataset.

Parameters and Conditions	Specification
Welding processes	MAG, MIG and SAW
Base materials	ST37 carbon steel, Hardox 450 steel and aluminum
Welding mode	Synergic S-Pulse
Welding current	136–278 A
Welding voltage	22.9–32.2 V
Welding travel speed	373–716 mm/min
Imaging device	TCL 20 Pro 5G smartphone
Camera sensor	Sony IMX582, 48 MP
Camera aperture	f/1.79
Image stabilization	Optical image stabilization (OIS)
Autofocus system	Phase-detection autofocus (PDAF)
Lighting conditions	Existing industrial ambient lighting
Shooting distance	103–358 mm
Shooting angle	Approximately 0–15° relative to the surface normal

**Table 2:** Symbol definition for XAI methods.

Notation	Meanings
*N*	Total number of test samples
*i*	Sample index
$Y_{i}^{c}$	Confidence score of class *c* for the original image
$x_{i}$	Individual decrease value
μ	Mean decrease value
$O_{i}^{c}$	Confidence score after applying the Eigen-CAM explanation mask
*c*	Target class label ID

**Table 3:** System environment and hardware specifications.

Component	Specification
Operating System	Google Colab (Linux)
GPU	NVIDIA Tesla L4 (22 GB VRAM)
Python	3.12.13
PyTorch	2.10.0 + CUDA 12.8 (torch-2.10.0+cu128)
Ultralytics	v8.4.112

**Table 4:** Comparison of architectural complexity, computational cost, and inference time among the evaluated models.

Model	Pre-Trained	Params (M)	Flops (G)	IT (ms)
YOLO11-N	yolo11n	2.59	6.53	4.59
YOLO11-S	yolo11s	9.42	21.66	8.17
YOLO11-M	yolo11m	20.53	68.24	13.16
YOLO11-L	yolo11l	25.41	87.35	16.43
YOLO11-X	yolo11x	56.94	195.47	27.82
CAEFRER-YOLO11	yolo11l	39.11	108.85	19.17
RT-DETR-L	rtdetr-l	32.86	109.92	20.30
RT-DETR-X	rtdetr-x	67.32	234.60	31.65

**Table 5:** Performance comparison of different algorithms based on mAP metrics and mAR metric.

Model	${\mathrm{mAP}}_{50–95}$ (%)	${\mathrm{mAP}}_{s}$ (%)	${\mathrm{mAP}}_{m}$ (%)	${\mathrm{mAP}}_{l}$ (%)	${\mathrm{mAR}}_{100}$ (%)
YOLO11-N	49.85±1.22	28.94±1.57	49.99±0.97	60.82±2.95	55.75±1.43
YOLO11-S	51.74±1.78	31.77±1.91	51.45±1.22	62.68±2.16	56.17±1.28
YOLO11-M	52.13±1.65	34.77±2.19	49.06±1.57	61.12±2.08	56.71±1.39
YOLO11-L	53.64±1.84	34.64±2.81	52.39±0.81	63.29±1.73	57.25±0.91
YOLO11-X	52.09±1.43	36.70±2.25	50.38±1.35	61.64±2.51	56.89±1.24
**CAEFRER-YOLO11**	55.73±1.58	38.02±2.10	54.77±0.75	64.10±1.55	59.03±1.49
RT-DETR-L	54.98±1.47	35.85±2.33	54.12±1.08	65.95±1.74	60.15±1.09
RT-DETR-X	54.26±1.76	34.97±2.59	54.37±1.24	62.78±2.26	59.45±1.45

**Table 6:** Class-wise performance comparison under different dataset sizes.

Classes	Dataset Size	Model	Precision (%)	Recall (%)	${\mathrm{AP}}_{50}$ (%)
Class 0	1437	YOLO11-L	82.07±2.32	77.91±2.71	75.11±2.64
		Ours	84.74±1.44	81.54±1.97	81.36±2.25
		RT-DETR-L	81.13±1.67	83.82±1.80	76.80±1.79
	3510	YOLO11-L	89.46±2.40	84.91±2.02	86.12±2.16
		Ours	90.73±1.94	87.65±1.50	91.49±1.71
		RT-DETR-L	88.13±1.46	86.84±2.26	87.60±1.85
Class 1	1437	YOLO11-L	77.54±1.31	57.58±1.67	63.64±2.14
		Ours	79.49±1.77	61.47±1.54	63.29±1.56
		RT-DETR-L	78.21±2.29	64.27±1.09	65.92±1.63
	3510	YOLO11-L	88.23±1.72	81.65±1.82	83.51±2.94
		Ours	88.95±0.99	82.39±1.92	84.19±2.12
		RT-DETR-L	90.36±1.36	83.91±2.15	85.52±1.97
Class 2	1437	YOLO11-L	88.78±2.09	87.30±2.76	90.30±1.65
		Ours	91.98±1.68	89.77±1.33	91.45±2.17
		RT-DETR-L	92.42±1.41	89.32±1.71	92.87±2.80
	3510	YOLO11-L	91.19±1.01	89.07±2.57	87.66±2.03
		Ours	93.01±0.82	89.49±1.40	90.14±2.34
		RT-DETR-L	91.50±1.24	90.82±1.24	89.78±1.81

**Table 7:** Summary statistics of the 10-fold cross-validation results for CAEFRER-YOLO11.

Metrics	Mean	Std. Dev.	95% CI	Coefficient of Variation (%)
${\mathrm{mAP}}_{50}$	0.8875	0.0263	[0.8687,0.9063]	2.97
${\mathrm{mAP}}_{50–95}$	0.5006	0.0222	[0.4847,0.5165]	4.43

**Table 8:** Ablation study and baseline comparison of CAEFRER-YOLO11 across three random seeds (25, 30, and 42).

Model	Backbone	Neck	Head	P3 Enhanced	P4 Enhanced	P5 Enhanced	${\mathrm{mAP}}_{50}$ (%)
Model 1	YOLO11	YOLO11	YOLO11	–	–	–	85.76±2.37
Model 2	RT-DETR	RT-DETR	RT-DETR	–	–	–	87.63±2.56
Model 3	YOLO11	YOLO11	RT-DETR	*√*	–	–	87.51±1.76
Model 4	YOLO11	YOLO11	RT-DETR	–	*√*	–	86.72±2.34
Model 5	YOLO11	YOLO11	RT-DETR	*√*	*√*	–	88.65±2.11
**Model 6 (Ours)**	YOLO11	YOLO11	RT-DETR	*√*	*√*	*√*	88.94±1.89

**Table 9:** Quantitative evaluation of XAI-based activation responses under M1 and M2 masking conditions across all test samples.

Model	M1		M2	
	AD (%)	SDD (%)	AD (%)	SDD (%)
YOLO11-L	7.94	9.41	5.08	8.65
CAEFRER-YOLO11	9.23	7.40	−1.63	5.24
RT-DETR-L	8.67	10.03	6.25	7.31

## Data Availability

The data presented in this study are openly available in Zenodo at https://doi.org/10.5281/zenodo.23468201.

## References

[1] Bala Y., Rajendran D.K. (2024). Global welding market growth. Automation in Welding Industry: Incorporating Artificial Intelligence, Machine Learning and Other Technologies.

[2] Yan X., Lian X., Ban J., He W., Deng S., Yang W., Chen D. (2025). Degradation heterogeneity in active X70 pipeline welds microstructure-property coupling under multiphysics environments of hydrogen-blended natural gas. Processes.

[3] Wang Q., Cao S., Xiang S., Zhang D., Song T., Xu Z., Jiang Q. (2026). Weld width measurement and defect detection method for desiccant cartridges based on machine vision. Sensors.

[4] Tang W., Zhu W. (2025). Application of artificial intelligence in predicting weld quality and defects. IEEE Access.

[5] Hrvoje C., Patrik V., Viktor L., Matija G., Maja T. (2026). Robotic welding technologies for intersecting and irregular pipes and pipe joints toward automated production line integration: A review. Appl. Sci..

[6] Senthamilarasi C., Anbarasi M.P., Vinod B., Kumar K.S., Nagalaxman G. (2026). Hybrid machine learning models for automated classification of weld defects in gas metal arc robotic welding. Discov. Artif. Intell..

[7] Zhang J., Li P.H., Zhang M.X., Shen K.H., Xing Y.Z. (2026). A review of algorithms for surface defect detection in welds based on machine learning. Expert Syst. Appl..

[8] Dilbaz A., Ozkan I.A. (2025). Simulation-based spot welding inspection on automotive chassis using YOLO-powered image processing. Int. J. Automot. Eng. Technol..

[9] Zhang R., Pan C., Chen Z., Fan J., Chen Z., Yuan B. (2026). An improved lightweight YOLOv11 algorithm for weld surface defect detection. Sci. Rep..

[10] Qi J., Xu Z., Cheng L., Liu S., Zhong K., Ai D. (2026). Welding image defect detection based on YOLO11. Anti-Corros. Methods Mater..

[11] Pernando Y., Andryani N.A.C., Chowanda A., Budiharto W. (2025). Low-light welding defect detection using Retinex-enhanced YOLOv11 for steel welding inspection. IEEE 2025 International Conference on Communication, Networks and Satellite (COMNETSAT).

[12] Liu G., Yang D., Ye J., Lu H., Wang Z., Zhao Y. (2025). A real-time welding defect detection framework based on RT-DETR deep neural network. Adv. Eng. Inform..

[13] Kwon J.E., Park J.H., Kim J.H., Lee Y.H., Cho S.I. (2023). Context and scale-aware YOLO for welding defect detection. NDT E Int..

[14] Wu K., Song L., Feng S., Wu Q., Huang J., Sun T., Liu P. (2025). LA-YOLO: A lightweight and accurate deep learning model for welding defect detection. Weld. Int..

[15] Zhao H., Chen J., Xie J. (2024). Using multi-scale detection mechanism and dynamic monitor defect area for pipeline weld identification. IEEE 2024 6th International Conference on Intelligent Control, Measurement and Signal Processing (ICMSP).

[16] Yang H., Wang H., Li H., Song X. (2022). Weld defect cascaded detection model based on bidirectional multi-scale feature fusion and shape pre-classification. ISIJ Int..

[17] Dilbaz A., Ozkan I.A., Özçelik Z. (2025). Simulation-based optimization of robotic weld seam tracking using fuzzy C-means clustering and PID control. Duzce Univ. J. Sci. Technol..

[18] Mobaraki M. (2025). Vision-Based Seam Tracking and Multi-Modal Defect Detection in GMAW Fillet Welding Using Artificial Intelligence. Ph.D. Thesis.

[19] Muhammad M.B., Yeasin M. (2020). Eigen-CAM: Class Activation Map using Principal Components. IEEE 2020 International Joint Conference on Neural Networks (IJCNN).

[20] Yang B., Liu S., Wang X., Shi L. (2026). A welding defect detection network based on multi-dimensional collaborative enhancement of spatial-frequency features. Nondestruct. Test. Eval..

[21] Liang Y., Yu B., Ding M., Hu W., Jin Y., Yuan Y. (2025). WELD-DETR: A real-time welding defect detection framework with multi-scale feature fusion and multi-kernel perception optimization. Sensors.

[22] Eren B. (2026). Deep learning approaches for weld defect detection: A comprehensive review of models, applications, and future directions. Comput. Ind. Eng..

[23] Block S.B., da Silva R.D., Lazzaretti A.E., Minetto R. (2024). LoHi-WELD: A novel industrial dataset for weld defect detection and classification, a deep learning study, and future perspectives. IEEE Access.

[24] Zhang X., Zheng Q., Zhu P., Ma W. (2025). INWELD—An industrial dataset for object detection and instance segmentation of weld images in production scenarios. Appl. Sci..

[25] Wang G.Q., Zhang C.Z., Chen M.-S., Lin Y.C., Tan X.H., Liang P. (2026). Yolo-MSAPF: Multiscale alignment fusion with parallel feature filtering model for high accuracy weld defect detection. IEEE Trans. Instrum. Meas..

[26] Luo Y., Ling J., Wang J., Zhang H., Chen F., Xiao X., Lu N. (2025). SFW-YOLO: A lightweight multi-scale dynamic attention network for weld defect detection in steel bridge inspection. Measurement.

[27] Rappe N.A., Kirda A.W., Yassin H., Bartoszuk M., Caesarendra W. (2025). Application of YOLOv8 in fusion welding defect detection on carbon steel for potential remote visual inspection. Adv. Sci. Technol. Res. J..

[28] Celles A.K.M., Leviste L.R.A., Lim K.H.W., Santeco E.S., Comia L.V. (2026). A deep vision segmentation framework for weld defect detection and structural integrity analysis. Proceedings of the 8th International Conference on Intelligent Sustainable Systems (ICISS).

[29] Meng D., Chen X., Fan Z., Zeng G., Li H., Zhou B. (2021). Conditional DETR for fast training convergence. IEEE/CVF International Conference on Computer Vision (ICCV).

[30] González Hernández A.J., Sánchez Hernández J.P., Hernández Rabadán D.L., Frausto Solis J., González Barbosa J. (2025). An analysis of YOLO models versus RT-DETR applied to multi-object detection in images. Int. J. Comb. Optim. Probl. Inform..

[31] Dilbaz A. (2026). YOLOv10 and RT-DETR for weld defect detection: Experimental evaluation of dataset splits and explainability. Int. J. Appl. Methods Electron. Comput..

[32] Zhang M., Hu Y., Xu B., Luo L., Wang S. (2025). DSF-YOLO for weld defect detection in X-ray images with dynamic staged fusion. Sci. Rep..

[33] López G., Ramírez P.D., Vega E., Pizarro F., Toro J., Parra C. (2025). WeldVGG: A VGG-Inspired Deep Learning Model for Weld Defect Classification from Radiographic Images with Visual Interpretability. Sensors.

[34] Wu X., Liu C., Zhang H., Xu P. (2025). ADFE-DET: An adaptive dynamic feature enhancement algorithm for weld defect detection. Appl. Sci..

[35] Liu T., Zheng H., Bao J., Zheng P., Wang J., Wang L., Zhong R.Y. (2022). An explainable laser welding defect recognition method based on multi-scale class activation mapping. IEEE Trans. Instrum. Meas..

[36] Nikam D., Nikam S., Bhosale T., Harkin D., Sawant M., McGarrigle C. (2026). Deep CNN-based multi-class TIG welding defect classification using HDR images with explainable AI. J. Manuf. Mater. Process..

[37] Redmon J., Divvala S., Girshick R., Farhadi A. (2016). You only look once: Unified, real-time object detection. Proceedings of the IEEE Conference on Computer Vision and Pattern Recognition (CVPR).

[38] Liu N., Kong W., Jiang Y., Shan W., Xiao H., Li X. (2026). EdgeEnhance-YOLO: A lightweight small object detection model with multidimensional edge enhancement. IEEE Trans. Aerosp. Electron. Syst..

[39] Li Z., Li S., Li W., Liu M., Li S., Chen X. (2026). WID-YOLO11: Weld surface defect detection via frequency-domain feature decomposition and multi-scale dilated attention. Information.

[40] Zhou Z., Ge Q., Zhang M., He X., Wang K. (2026). MEFPNet: A multi-scale enhanced feature pyramid network for similarity-confounded substation surface-defect detection. Neurocomputing.

[41] Vaswani A., Shazeer N., Parmar N., Uszkoreit J., Jones L., Gomez A.N., Kaiser Ł., Polosukhin I. (2017). Attention is all you need. Advances in Neural Information Processing Systems (NeurIPS).

[42] Huang J., Kane A., Zhou F., Wei Y., Shi H. (2026). Revisiting real-time detection transformer with efficient encoder design. Proceedings of the IEEE/CVF Conference on Computer Vision and Pattern Recognition (CVPR) Findings.

[43] Ding X., Zhang X., Ma N., Han J., Ding G., Sun J. (2021). RepVGG: Making VGG-style ConvNets Great Again. Proceedings of the IEEE/CVF Conference on Computer Vision and Pattern Recognition (CVPR).

[44] Shi D., Zhao C., Shao J., Feng M., Luo L., Ouyang B., Huang J. (2025). Context-Aware Enhanced Feature Refinement for small object detection with Deformable DETR. Front. Neurorobot..

[45] Wijaya S.A. (2025). Weld Defect Detection Dataset. Kaggle. https://www.kaggle.com/datasets/sukmaadhiwijaya/weld-defect-detection-dataset.

[46] Feng J., Wang J., Zhao X., Liu Z., Ding Y. (2024). Laser weld spot detection based on YOLO-Weld. Sci. Rep..

[47] Zhang W., Zhang H., Zhang X., Shen X., Zou L. (2025). IP-CAM: Class activation mapping based on importance weights and principal-component weights for better and simpler visual explanations. Comput. Vis. Image Underst..

